# Supplementary material for: Neural networks differentiate between Middle and Later Stone Age lithic assemblages in eastern Africa
Source: PLoS One. 2020 Aug 26;15(8):e0237528. doi: 10.1371/journal.pone.0237528 (PMC7449415; doi:10.1371/journal.pone.0237528)
Supplement: S1 File — (DOCX) [file pone.0237528.s001.docx]

The data set used in the analyses was produced from an extensive literature review, focusing on Middle Stone Age (MSA) and Later Stone Age (LSA) sites from eastern Africa that are reliably associated with chronometric dates spanning the period of Marine Isotope Stage (MIS) 5 (130-71 thousand years ago [ka]) to MIS 2 (28-11.5ka). Only assemblages that were attributed to either MSA or LSA were included, with a small number of additional assemblages listed as transitional or MSA/LSA (e.g. Kiese II 12-17 [1]) excluded. The full list of sites and assemblages included in the analysis is presented in Table SI1, and their distribution illustrated in Figures SI1-3, split between LSA, MSA 3/4, and MIS 5. A dataset of all artefact types reported was compiled.

Table SI1: Sites and assemblages used in the analysis, indicating the group (LSA, MIS3/4 MSA, MIS5 MSA) they contribute to and key references.

|  | **Site** | **Assemblage** | **Reference** |
| --- | --- | --- | --- |
| **LSA** | Aladi Springs | LSA | [2] |
|  | Enkapune ya Muto | DBL; GG | [3] |
|  | Kiese II | 3; 4; 5; 6; 7; 8; 9; 10; 11 | [1] |
|  | Lukenya Hill | GvJm22 E120_150 | [4] |
|  | Lukenya Hill | GvJm16_B | [5] |
|  | Mumba | Mumba_M_III_77 | [6] |
|  | Munyama Cave | Munyama Cave | [7] |
|  | Naisiusiu | 1931; 1969insitu; 1972 | [8] |
|  | Nasera | Nasera_4_5 | [6] |
|  | Pange ya Saidi | 5; 6; 7&8; 9; 10; 11; 1213; 14; 15; 16 | [9] |
| **MIS3/4 MSA** | Enkapune ya Muto | RBL4 | [10] |
|  | Fincha Habera | 8_8; 8_9; 8_10; 8_11; 9 | [11] |
|  | Goda Butchia | GodaButchia70_110 | [12] |
|  | Karungu | Kisaaka_Main | [13] |
|  | Kiese II | 18; 19; 20; 21 | [1] |
|  | Laas Geel | LaasGeel_SU_711 | [14] |
|  | Lukenya Hill | GvJm22_F170_205 | [4] |
|  | Lukenya Hill | GvJm46 | [10][15] |
|  | Magubike | MSA | [16] |
|  | Mochena Borago | LowerT; UpperT; RGroup; SGroup | [17] |
|  | Mumba | L_III_38; L_V_81; MU_V_81; U_V_38; U_VI_A; L_VI_38 | [6] |
|  | Nasera | 6_7; 8/9_11; 12_17 | [6] |
|  | Rusinga | Nyamita | [18] |
|  | Shurmai | MSA | [19] |
| **MIS5 MSA** | Abdur | Abdur_N_C_S | [20] |
|  | Aduma | A1; A4C; A5Ex; A5ExSurf; A8; A8AC; A8Ag; A8ASurf; A8B; VP1/1; VP1/3 | [21] |
|  | Eyasi Shore | 77_81; N_Surface; W_InSitu; W_Surface | [6] |
|  | KapedoTuffs |  | [22] |
|  | Karungu | A3Ex; Kisaaka_ZTG | [13] |
|  | Marmonet Drift | H4; H5 | [3] |
|  | Mumba | L_VI_A; VI_B | [6] |
|  | Omo | BNS_L3; BNS<50m | [23] |
|  | Pange ya Saidi | 17; 18; 19 | [9] |
|  | VictoriaCabera | 2; 2a | [24] |


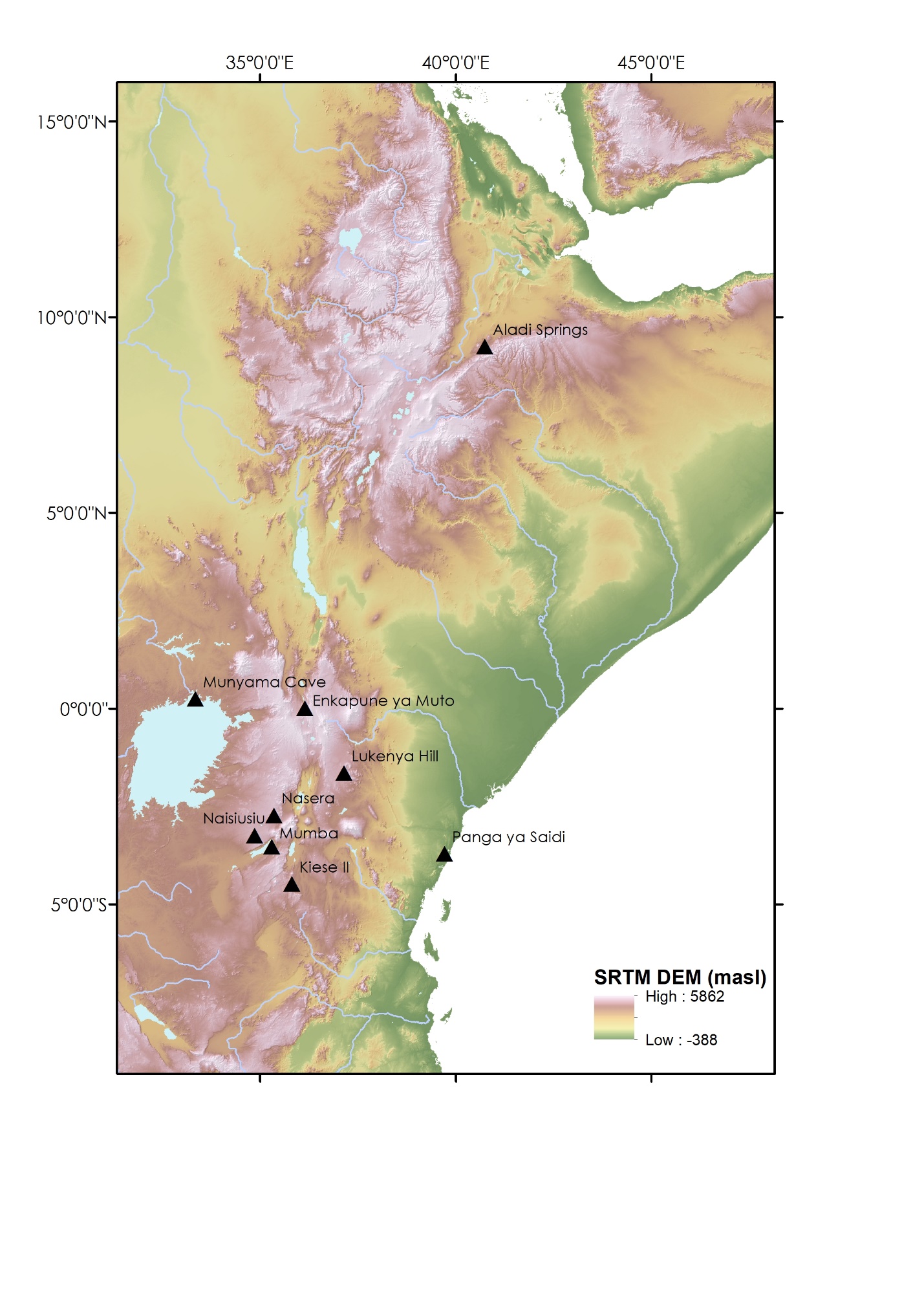


Figure SI1: Distribution of LSA sites included in the analysis.


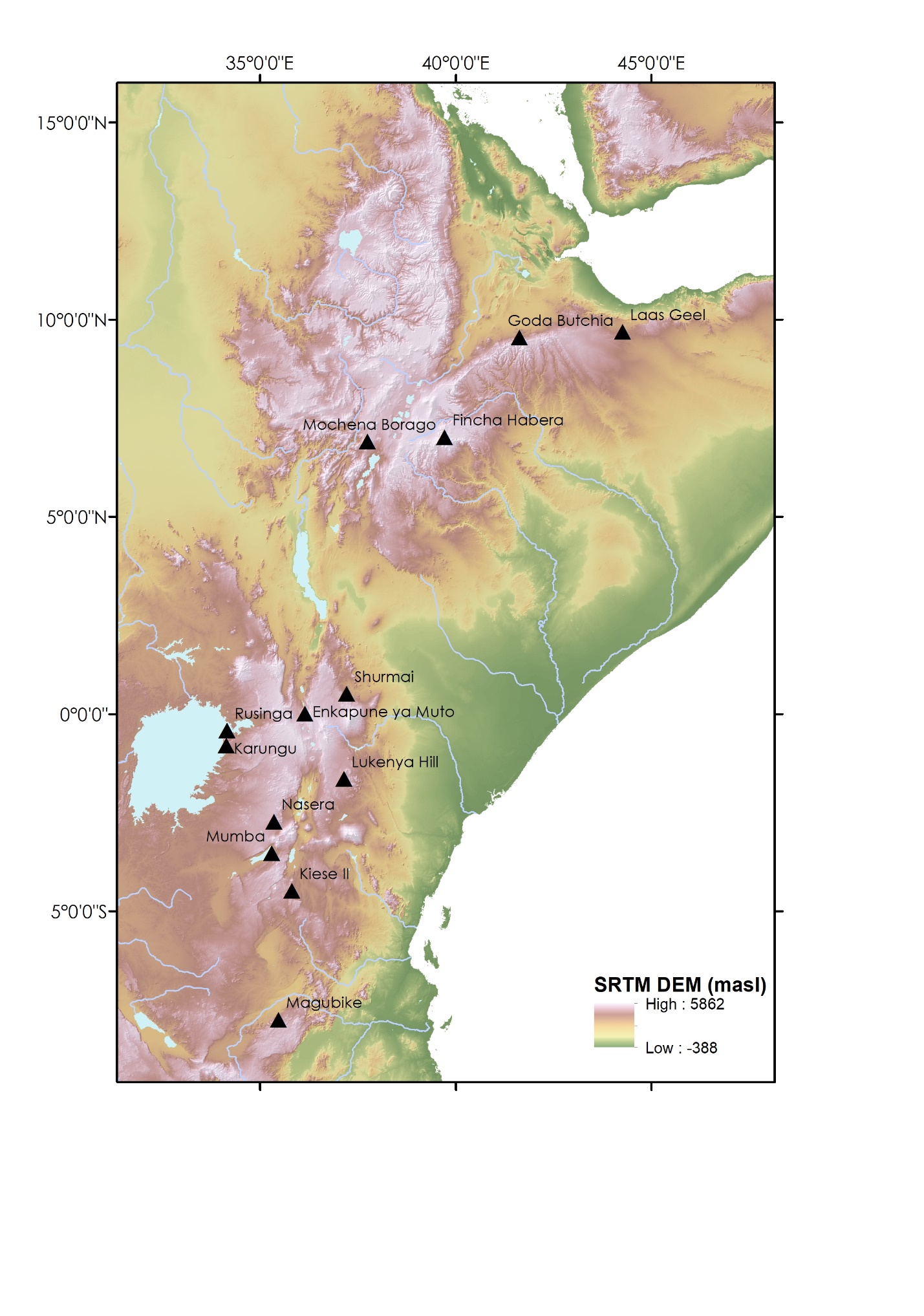


Figure SI2: Distribution of MIS 3 & 4 MSA sites included in the analysis.


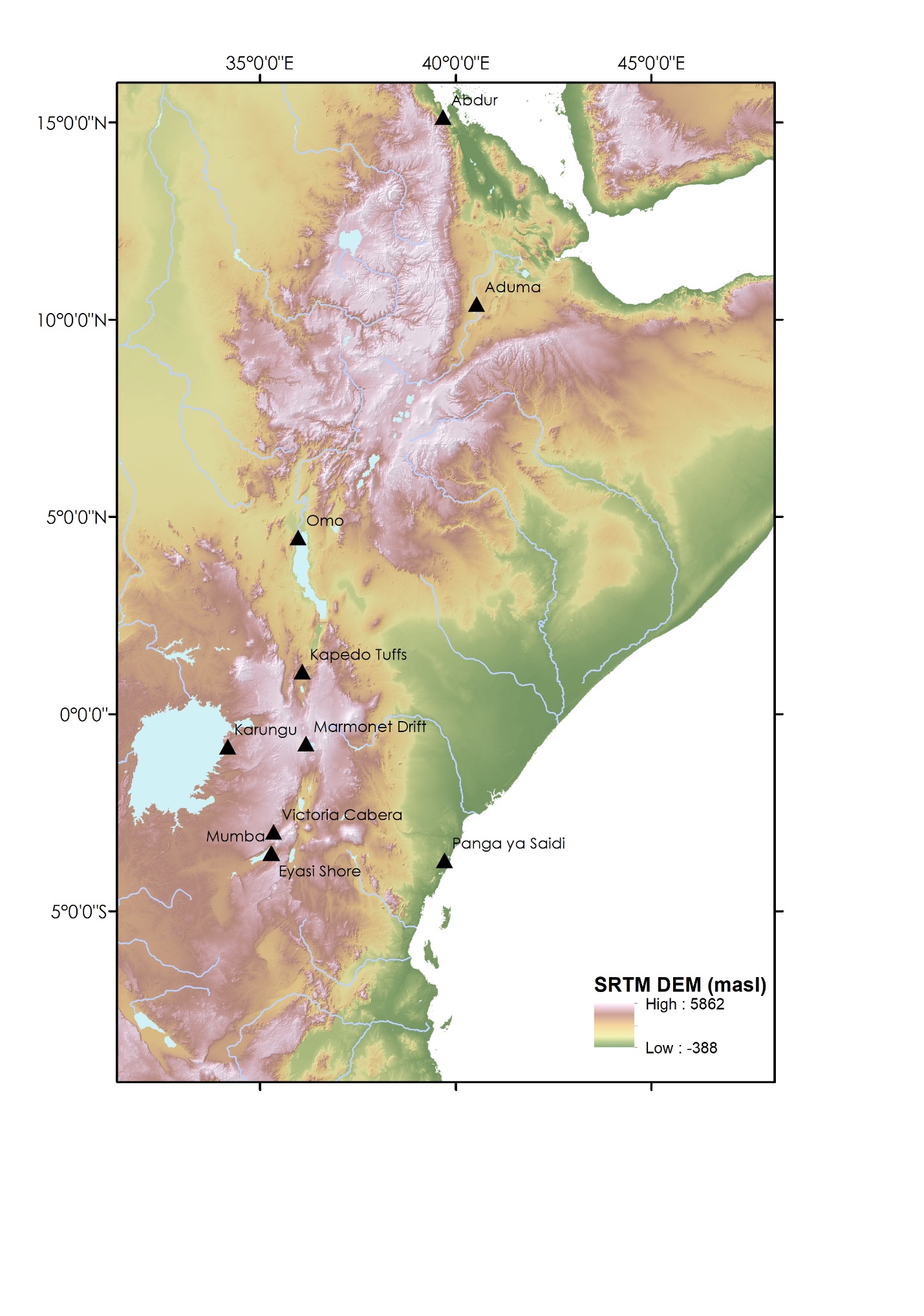


Figure SI3: Distribution of MIS 5 MSA sites included in the analysis.

This initial dataset indicated the presence of considerable variability in terms have been used to describe stone artefacts from eastern Africa, and a number of steps were employed to compress these data into a smaller range of artefact types. All types that lack the means to resolve meaningful differences in assemblage composition or were ubiquitous (e.g. flakes; debris; unspecified cores or unspecified retouched tools) are not considered here. In many instances, some researchers have reported more detailed types (e.g. side and end scraper) compared to others (e.g. scraper), and it has been necessary to adopt the lower resolution terminology. Following [25], we have combined diverse evidence for particular reduction strategies to be indicative of the presence of the broader technology. For instance, the presence of Levallois flake cores, Levallois flakes, and retouched Levallois flakes all indicate the application of Levallois Flake Technology, and the presence of one element may serve as an index of this reduction strategy. In rare instances, pairs of functionally comparable types have been combined, such as awls, borers and drills. Finally, for inclusion within the final dataset, artefact forms were required to occur in at least 5 different assemblages, to prevent rare types skewing the analysis, and each assemblage was required to present at least 2 of the retained artefact types, such that the analysis can examine constellations of co-occurrence or mutual exclusivity. The final range of artefact forms includes: Backed Pieces, Bipolar Technology, Blade Technology, Borers, Burins, Centripetal Technology, Core Tool, Denticulate, Levallois Blade Technology, Levallois Flake Technology, Levallois Point Technology, Notch, Platform Core, Point Technology, RT Bifacial and Scraper. These broadly reflect comparable categories employed by other syntheses of eastern African MSA and LSA ([10]; [26]; [27]). The presence and absence of these artefact forms is illustrated in Figure SI4 differentiating LSA, MIS 3/4 MSA and MIS 5 MSA assemblages. Below, the different terms are briefly discussed, alongside presenting the range of terminology used by the reporting researchers that have been combined.


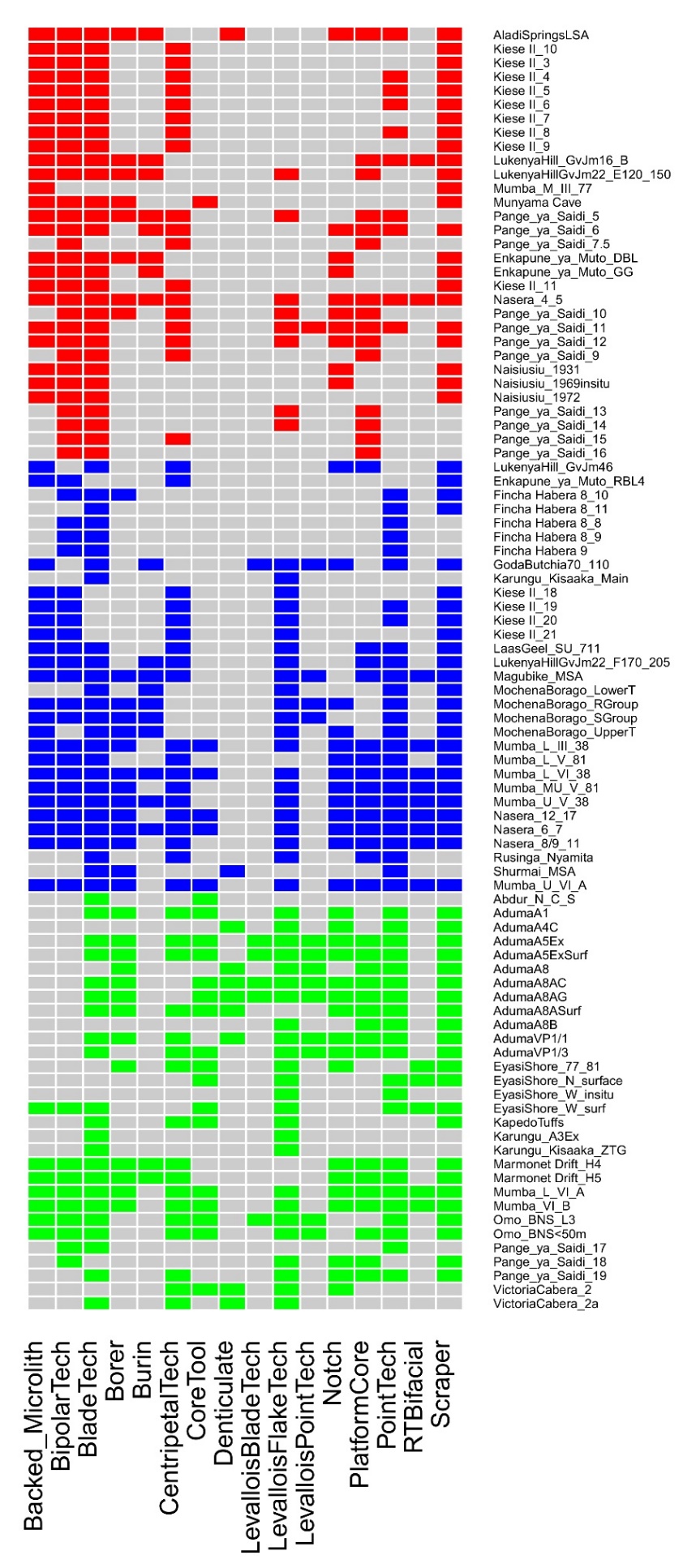


Figure SI4: Presence/absence matrix of 16 artefact forms used in the analysis illustrating the presence of each artefact form with LSA (red), MIS 3 and 4 MSA (blue) and MIS 5 MSA (green) assemblages, with absences shown in grey.

**Backed Pieces**

Backing is a distinct form of retouching that produces an abrupt edge, with the blunted edge either facilitating application of force as a hand-held tool or providing a surface for hafting within a composite tool. The majority of assemblages in which Backed Pieces are identified as present directly use the term backed. A small number of other types are included which are identified as specific shapes of backed pieces (e.g. Triangles) or as alternate terms that are broadly synonymous with the use of Backed Pieces (e.g. Composite Tools). Table SI2 lists all terms that have been included in the category Backed Pieces.

Table SI2: Artefact terms used within the literature that are included as Backed Pieces

| **Term** |
| --- |
| Assymetric backed |
| Backed |
| Backed Blades |
| backed blades sloped down at 1 end |
| backed blades sloped down at 2 ends |
| backed blades sloped down at 2 ends with utilisation |
| Backed Flakes |
| Backed knife |
| Backed Microliths |
| Backed Piece |
| Backed Piece Fragment |
| Backed: crescents |
| Backed: curved backed blades |
| Backed: curved backed microliths |
| Backed: double backed blades |
| Backed: other sundries |
| Backed: psuedocrescents |
| Backed: straight backed baldes |
| Backed: trapezes |
| Backed: trapezoids |
| Backed: triangles |
| Backed: truncated blades oblique |
| Backed: truncated blades orthogonal |
| Backed: truncated blades side and oblique |
| Combination Tools |
| Composite tools |
| Crescent |
| curve backed |
| double-backed blades |
| geometric |
| Microliths |
| misc backed pieces |
| Pointed backed bladelets |
| straight backed |
| straight-backed blades |
| Total microliths |
| Trapezoid |
| triangle |
| Triangle |
| Truncations |
| Backed bladelets |
| broken crescents and trimmed bladelets |

**Bipolar Technology**

Bipolar Technology relates to the use of a hammer and anvil to remove flakes from a core, resulting in characteristic crushing of platform surfaces, two percussion points on cores and the resulting double bulb of percussion marks on flake ventral surfaces. Prior to the widespread recognition of this technological approach, some researchers considered bipolar cores to have been retouched as tools and have been reported as Outil Escaille, Pieces Escaille or Scaled Pieces. Table SI3 lists all terms that have been included in the category Bipolar Technology.

Table SI3: Artefact terms used within the literature that are included as Bipolar Technology

| **Term** |
| --- |
| Bipolar |
| Bipolar Core |
| Bipolar Flake |
| Bipolar Redirecting Flake |
| BipolarCore |
| Outil Escaille |
| Outils ecasilles cum core |
| Outils ecasilles fragment |
| Outils ecasilles Plain |
| Pieces ecaillees |
| Platform alternate ended/Bipolar |
| Scalar Pieces |
| Scaled Pieces |
| Used Bipolar Blade |
| used Bipolar Blade Frag |
| Used Bipolar Bladelet |
| Used Bipolar Flake |
| Used Bipolar Flake Frag |
| Utilized retouched bipolar flake |
| platf/bipolar |

**Blade Technology**

Amongst stone tool assemblages, blades are typically considered flake blanks where the length is at least double the width of the piece, and Blade Technology is used here to combine evidence for dedicated blade production strategies evident either from cores showing blade removals, or the presence of blades themselves. No differentiation is made between blades and bladelets as no single standard definition is used across the literature. Table SI4 lists all terms that have been included in the category Blade Technology.

Table SI4: Artefact terms used within the literature that are included as Blade Technology

| **Term** |
| --- |
| Backed bladelets |
| broken crescents and trimmed bladelets |
| Backed Blade |
| Blade |
| blade and frag |
| Blade Core |
| Blade Fragments |
| Blade Tools |
| blade, denticulate |
| blade, retouched |
| bladelents, trimmed one end |
| Bladelet |
| Bladelet production |
| Bladelet tools |
| bladelet, retouched |
| bladelets, both edges blunted |
| bladelets, straight one edge blunted |
| bladelets, trimmed both ends |
| blades, boken |
| blades, edge chipped or blunted by use |
| blades, edges trimmed (broken) |
| blades, sharp edged |
| comp blade |
| core, attempt blade |
| core, blade |
| core, bladelet |
| double side scraper blade |
| Edge Nibbled Blade |
| flake/blade frags |
| flakes/blades |
| frag blade |
| Medial/Distal Blade |
| Medial/Distal DPS Blade |
| Non-Levalloisblade Frag |
| obliquely truncated blade |
| platform core suggesting blade production |
| Platform Prismatic/Blade |
| PrismaticCore |
| Retouched blades |
| RT Blade |
| RT Blade Frag |
| Single platform blade |
| Single platform bladelet |
| single side scraper blade |
| Truncated Blade |
| Used Blade |
| Used Blade Frag |
| Used RT Blade |
| Used RT Blade Frag |
| utilised blades and flakes |
| Whole/Prox Blade |
| Endscraper on blades |

**Borer**

Borers are retouched tools which have been shaped to have a robust, thick point, and are typically associated with drilling and piercing activities, with a number of different terms indicating similar forms and functions. Table SI5 lists all terms that have been included in the category Borer.

Table SI5: Artefact terms used within the literature that are included as Borer

| **Term** |
| --- |
| Bec |
| Borer |
| drills/awls/becs |
| Drills/borers |
| fabricators |
| Graver |
| Percoirs |
| perforator/borer |
| Perforators |

**Burin**

Burination is a technological distinct form of retouch in which small retouch flakes or burin spalls are removed from the edge of a flake, preserving part of both the dorsal and ventral surface. Table SI6 lists all terms that have been included in the category Burin.

Table SI6: Artefact terms used within the literature that are included as Burin.

| Term |
| --- |
| Backed: krukowski microburins |
| Backed: microburins |
| Burin |
| Burin Spall |
| Burins on truncation |
| Dihedral burins |

**Centripetal Technology**

Patterns of centripetal or radial flaking of prepared cores are widely reported in eastern Africa, though diverse terms have been used to reflect this. Such cores typically display a distinct flaking and platform surface with a pattern of flaking that works around the interface of the two and flaking into the centre of the core, resulting in diagnostic dorsal scar and flake scare patterns. Here, we combine evidence for centripetal or radial flaking and discoidal reduction schemes, including the production of pseudo-Levallois points as a key product of the latter under the term Centripetal Technology. Notably, there was no co-occurrence of the use of key terms ‘Centripetal’, ‘Discoidal’ and ‘Radial’ for cores or flakes amongst the dataset, and the absence of the co-use of these terms suggests that they have been used synonymously. Table SI7 lists all terms that have been included in the category Centripetal Technology.

Table SI7: Artefact terms used within the literature that are included as Centripetal Technology.

| **Term** |
| --- |
| Asymmetrical discoid |
| Bifacial Centripetal Core |
| Centripetal Core |
| Centripetal Flake (Discoid) |
| Centripetal Flaking |
| core, discoid partial |
| core, discoidal |
| DiscCore |
| Discoidal |
| Discoidal Core |
| Partial Discoid |
| Part-peripheral |
| Peripheral Core |
| platf/perhiperal |
| Prepared Core |
| Pseudo-Levallois point |
| PseudolevalloisPoint |
| Radial Core |
| Radial/disc |
| Unifacial Centripetal Core |
| Unifacial Discoid |

**Core Tools**

A range of large core tools, as opposed to retouched tools on flakes, were identified in the synthesis of the dataset, though no single form appeared at a frequency large enough to be included individually. Nevertheless, this indicated the repeated occurrence of core tools which we represent through the grouping of Core Tools. Table SI8 lists all terms that have been included in the category Core Tools.

Table SI8: Artefact terms used within the literature that are included as Core Tools

| **Term** |
| --- |
| Bifacial Chopper |
| Bifacial Handaxes |
| Chopping Tool |
| Core Axes |
| Core scrapers |
| core, chopper |
| Core-Axe |
| Heavy duty |
| Heavy Duty |
| minimally retouched bifacial pick |
| Ovates |
| scraper, core |

**Denticulate**

Denticulates are retouched tools with distinctly serrated edges. Although widely acknowledged as a form of tool, denticulates may occur on a spectrum of reduction intensity, marking more intensive use of the tool than lightly retouched tools which have not significantly impacted the shape of a working edge (e.g. scraper). Table SI9 lists all terms that have been included in the category Denticulate.

Table SI9: Artefact terms used within the literature that are included as Denticulate

| **Term** |
| --- |
| Denticulate |
| Denticulated pieces |
| Denticulate-Endscraper |
| flake, denticulate |

**Levallois Blade, Flake and Point Technology**

Levallois technologies involve preparing cores in particular ways, including a volumetric hierarchy of a bifacial core with distinct prepared platform surface and controlling flaking surface convexities, in order to remove blanks of a predetermined shape and size. Reporting of Levallois technologies across eastern Africa for the MSA and LSA is varied in terms of detail, with few sites resolving clearly between preferential or recurrent flaking approaches, or patterns of flaking surface preparation and exploitation (e.g. unidirectional, centripetal). Some distinction is made within the data between the shape of blanks being produced, enabling differentiation of Levallois blade and point approaches. Where the blank shape has no been specified, presence of Levallois technology has been attributed to Levallois Flake approaches. Tables SI10, SI11 and SI12 lists all terms that have been included in the category Levallois Blade Technology, Levallois Flake Technology, and Levallois Point Technology.

Table SI10: Artefact terms used within the literature that are included as Levallois Blade Technology.

| **Term** |
| --- |
| core, Levallois blade |
| core, micro Levallois blade |
| flake/blade, Levallois rt |
| Levallois Blade |
| Levallois Blade Flake |

Table SI11: Artefact terms used within the literature that are included as Levallois Flake Technology.

| **Term** |
| --- |
| atypical levallois flake |
| comp levallois |
| core, Aduma |
| core, Levallois |
| core, Levallois approach |
| core, Levallois attempt |
| core, micro Aduma |
| core, micro Levallois |
| Endscrapers on Levallois blanks |
| frag levallois |
| Levallois |
| Levallois (method indeterminate) |
| levallois and frag |
| Levallois Bi |
| Levallois Cent |
| Levallois Core |
| Levallois Flake |
| Levallois Flake Core |
| Levallois Indet |
| Levallois Pref |
| Levallois preferential |
| Levallois Preferential Core |
| Levallois recurrent |
| Levallois technology |
| Levallois Uni |
| Levallois/Levalloisrelated |
| Levallois/Levalloisrelated Frag |
| LevalloisCore |
| pref recurrent levallois cores |
| Retouched Levallois flake or fragment |

Table SI12: Artefact terms used within the literature that are included as Levallois Blade Technology.

| **Term** |
| --- |
| atypical levallois point |
| core, Nubian |
| Levallois Point |
| Levallois Point (Blank) |
| Levallois Points (Blank) |
| point, acute tip |

**Notch**

Notches are retouched tools where retouching has formed a distinct concavity at the edge of the tool. Although widely acknowledged as a form of tool, notches may occur on a spectrum of reduction intensity, marking more intensive use of the tool than lightly retouched tools which have not substantially impacted the shape of a working edge (e.g. scraper). Table SI13 lists all terms that have been included in the category Notch.

Table SI13: Artefact terms used within the literature that are included as Notch.

| **Term** |
| --- |
| concave/notch |
| Notch |
| Notched Flake |
| Notched pieces |
| Notches/Denticulates |
| sinew frayers |

**Platform Core**

Flake cores with distinct prepared platforms are commonplace amongst eastern African MSA and LSA assemblages, and regularly accompany more formal core reduction strategies, and include reduction from single, opposed and multiple platforms. These core types were regularly differentiated from Amorphous/Assayed/Casual/Irregular/Opportunistic cores. Table SI14 lists all terms that have been included in the category Platform Cores.

Table SI14: Artefact terms used within the literature that are included as Platform Core.

| **Term** |
| --- |
| platf/bipolar |
| BidirectionalCore |
| core, multidirectional |
| core, single platform |
| Double platform (opposed) |
| Multi-PlatformCore |
| Opposed Platform Core |
| Platform Core |
| Platform Other |
| Platform Pyramidal |
| Platofrm Other |
| Polyhedron |
| Pyramidal |
| Single Platform Core |
| Single Platform Cores |
| Single platform prismatic |
| Unipolar |

**Point Technology**

Point blanks and retouched points are a common feature in the dataset. Given the nature of reporting assemblages did not always disambiguate points formed through debitage vs façonnage approaches, these have been combined here reflecting the focus on producing convergent artefact forms. Table SI15 lists all terms that have been included in the category Point Technology.

Table SI15: Artefact terms used within the literature that are included as Point Technology.

| Term |
| --- |
| Bifacial Point |
| foliate point |
| foliate point fragmenr |
| Other Points |
| other retouched point |
| Point |
| point |
| Point Flake |
| point fragment type |
| Point RT |
| Point with lateral retouch |
| Point with lateral retouches on both sides |
| point, blade |
| point, broken |
| point, classic MSA |
| point, damaged |
| point, misc. |
| point, short broad |
| point, small blunt |
| point/perforator |
| pointed piece |
| Points (RT) |
| RT Point |
| RT Point Frag |
| RT Utilised Point |
| scraper-point |
| Unifacial Point |
| Unifacial Point Frag |
| unifacially flaked points |
| Used Point |
| Used Point Frag |
| Used RT Point Frag |
| Used Rt Point/borer |

**Retouched Bifacial**

Bifacial retouching typically involves alternating retouch applied to both dorsal and ventral surfaces of a flake, as opposed to retouching of only one face. Table SI16 lists all terms that have been included in the category Retouched Bifacial.

Table SI16: Artefact terms used within the literature that are included as Retouched Bifacial.

| Term |
| --- |
| Bifacial Modified Pieces |
| Bifacial Piece |

**Scraper**

Scrapers are a commonplace retouched tool form where an edge has been systematically retouched along one or more of its edges to control its shape or refresh its cutting edge. At multiple sites, scrapers are distinguished from more informal retouching of flakes or used flakes. Scrapers may occur on a spectrum of reduction intensity, with more heavily used or retouched scrapers forming denticulated or notched edges and recorded as alternate tool forms as a result. Table SI17 lists all terms that have been included in the category Scraper.

Table SI17: Artefact terms used within the literature that are included as Scraper.

| **Term** |
| --- |
| Endscraper on blades |
| (End)Scraper |
| beaked and spurred scrapers |
| Casual Scraper |
| circ-oval scrapers |
| convergent sidescraper |
| convex end |
| convex side scraper |
| double scraper |
| double side and end scraper |
| double side scraper |
| End Scraper |
| Ensdscrapers on flakes |
| Formal Scraper |
| Hollow scraper |
| irregular scrapers |
| rect-oval scrapers |
| Scraper |
| Scraper Frag |
| Scraper Fragments |
| scraper, double side |
| scraper, end |
| scraper, end+side |
| scraper, mini |
| scraper, side |
| scraper, small convex |
| scraper, small non convex |
| scraper, tabular quartz |
| scraper, transverse |
| side and discoidal scraper |
| Side Scraper |
| simple end scrapers |
| single side scraper |
| small convex |
| sundry end |
| sundry side |
| thumbnail scraper |
| transverse scraper |
| triangular-trap. Scrapers |

Excluded Terms

A range of artefact forms reported in the literature were excluded from the dataset where they could not comprise a consistent grouping of forms apparent across at least five assemblages or where they were not considered to represent diagnostic patterns in lithic reduction and this terminology is reported in Table SI18.

Table SI16: Artefact terms that were excluded from the analysis.

| **Term** |
| --- |
| Amorphous Core |
| Angular Fragments |
| Angular Waste |
| Anvil |
| AssayedCore |
| AxeFlake |
| biface-thinning flake |
| blocky fragment |
| broken facteted flakes |
| Broken Flakes |
| Casual Core |
| casual/amorph |
| Chip |
| Chunk |
| CM Flake |
| Cobble |
| Cobble Frag |
| comp flake |
| complete flakes |
| Cordal Flake |
| Core |
| Core frag |
| Core Fragment |
| Core on flake |
| core rejuveneation flakes |
| Core trimmiing flakes and frags |
| core trimming element |
| core, amorphous |
| core, attempt |
| core, flat reversed |
| core, fragment |
| Cortical Flake no cortex platform |
| Debitage |
| DEBITAGE (chips chunks) |
| DebordantFlake |
| Debris |
| Derived Segment |
| divers/frag |
| edge damaged flakes and frags |
| Eraillure Flake |
| Facetted flakes |
| Flake |
| Flake & Flake Fragments |
| Flake Core |
| flake fragment other |
| Flake Fragments |
| Flake Tools |
| flake, retouched |
| Flaked Piece |
| flakes and spalls with beaks |
| flakes with bulbar thinning |
| Flaking debris |
| frag |
| grindstone |
| Grindstone Flake |
| grindstone? |
| Groundstone |
| hamerstone fragment |
| Hammerstone |
| Heat Pop Flake |
| Indet Core |
| Indet. |
| Informal Core |
| initial cortical flake |
| Intermediate |
| Irregular |
| Irregular Core |
| Irregular/other |
| Knife |
| Kombewa flake |
| Lithic Total |
| Measurable Flakes |
| Medial/Distal Flake |
| Misc retouched |
| Misc. Modified Pieces |
| modified |
| Naturally backed knives |
| nibbled/utilised |
| noncortical flake |
| Non-Levalloisflake |
| Non-Levalloisflake Frag |
| Opportunistic Core |
| ORDINARY FLAKES |
| Other |
| Other core |
| other/irregular |
| Pestle |
| Pestle Rubber |
| Polyhedral Core |
| Potlid Flake |
| pounding stone |
| PRF |
| proximal flake fragment |
| Redirecting Flake |
| residual cortical flake |
| Retouched Flake |
| Retouched Pieces |
| Retouchednon-Levalloisflakeorfragment |
| RT |
| RT Flake |
| Split Flake |
| StrikingPlatformPrepFlake |
| sundries |
| sundry cores |
| sundry flakes |
| Sundry Ground Stone |
| sundry modified |
| sundry retouched flakes |
| Tabular Core |
| tabular SDM |
| Tested Cobble |
| Tested Nodules |
| Tool Edge Fragment |
| Tool Fragments |
| Trimming RT Flake |
| Undetermined cores |
| Unipolat Core |
| unrt debitage |
| Unshaped Tools |
| Unspecified Core |
| Used Flake |
| Used Flake Frag |
| Used RT Chunk |
| Used RT Flake |
| Utilised Flakes |
| Utilized Debitage |
| Whole Flakes |
| Whole/Prox Flake |

References Cited:

1. Tryon CA, Lewis JE, Ranhorn KL, Kwekason A, Alex B, Laird MF, et al. Middle and later stone age chronology of kisese II rockshelter (UNESCO World Heritage Kondoa Rock-Art Sites), Tanzania. PLoS One. 2018;13: 1–24. doi:10.1371/journal.pone.0192029

2. Gossa T, Sahle Y, Negash A. A reassessment of the Middle and Later Stone Age lithic assemblages from Aladi Springs, Southern Afar Rift, Ethiopia. Azania Archaeol Res Africa. 2012;47: 210–222. doi:10.1080/0067270X.2012.676314

3. Slater PA. Change in lithic technological organization strategies during the Middle and Later Stone Ages in East Africa. 2016;10301922: 522. Available: https://search.proquest.com/docview/1857892080?accountid=14468%0Ahttp://wx7cf7zp2h.search.serialssolutions.com?ctx_ver=Z39.88-2004&ctx_enc=info:ofi/enc:UTF-8&rfr_id=info:sid/ProQuest+Dissertations+%26+Theses+Global&rft_val_fmt=info:ofi/fmt:kev:mtx:dissert

4. Tryon CA, Crevecoeur I, Faith JT, Ekshtain R, Nivens J, Patterson D, et al. Late Pleistocene age and archaeological context for the hominin calvaria from GvJm-22. Proc Natl Acad Sci. 2015;112: 2682–2687. doi:10.1073/pnas.1417909112

5. Merrick H V. Change in Late Pleistocene lithic industries in eastern Africa. University of California. 1975.

6. Mehlman MJ. Late Quaternary archaeological sequences in northern Tanzania. Univeristy of Illinois. 1989.

7. Van Noten F. Excavations at Munyama Cave. Antiquity. 1971;45: 56–58.

8. Leakey MD, Hay RL, Thurber DL, Protsch R, Berger R. Stratigraphy, Archaeology, and Age of the Ndutu and Naisiusiu Beds, Olduvai Gorge, Tanzania. World Archaeol. 1972;3: 328–341.

9. Shipton C, Roberts P, Archer W, Armitage SJ, Bita C, Blinkhorn J, et al. 78,000-year-old record of Middle and Later stone age innovation in an East African tropical forest. Nat Commun. 2018;9. doi:10.1038/s41467-018-04057-3

10. Basell LS. Middle Stone Age ( MSA ) site distributions in eastern Africa and their relationship to Quaternary environmental change , refugia and the evolution of Homo sapiens. Quat Sci Rev. 2008;27: 2484–2498. doi:10.1016/j.quascirev.2008.09.010

11. Ossendorf G, Groos AR, Bromm T, Tekelemariam MG, Glaser B, Schmidt J, et al. Middle Stone Age foragers resided in high elevations of the glaciated Bale Mountains, Ethiopia. Science (80- ). 2019;587: 583–587.

12. Pleurdeau D, Hovers E, Assefa Z, Asrat A. Cultural change or continuity in the late MSA / Early LSA of southeastern Ethiopia ? The site of Goda Buticha , Dire Dawa area. Quat Int. 2014;343: 117–135.

13. Faith JT, Tryon CA, Peppe DJ, Beverly EJ, Blegen N, Blumenthal S, et al. Paleoenvironmental context of the Middle Stone Age record from Karungu , Lake Victoria Basin , Kenya , and its implications for human and faunal dispersals in East Africa. J Hum Evol. 2015;83: 28–45. doi:10.1016/j.jhevol.2015.03.004

14. Gutherz X, Diaz A, Ménard C, Bon F, Douze K, Léa V, et al. The Hargeisan revisited: Lithic industries from shelter 7 of Laas Geel, Somaliland and the transition between the Middle and Late Stone Age in the Horn of Africa. Quat Int. 2014;343: 69–84. doi:10.1016/j.quaint.2014.04.038

15. Kelly AJ. Intra-Regional and Inter-Regional Variability in the East Turkana (Kenya) and Kenyan Middle Stone Age. State University of New Jersey. 1996.

16. Bushozi P. Lithic technology and hunting behaviour during the Middle Stone Age in Tanzania. University of Alberta. 2011.

17. Brandt S, Hildebrand E, Vogelsang R, Wolfhagen J, Wang H. Journal of Archaeological Science : Reports A new MIS 3 radiocarbon chronology for Mochena Borago Rockshelter , SW Ethiopia : Implications for the interpretation of Late Pleistocene chronostratigraphy and human behavior. J Archaeol Sci Reports. 2017;11: 352–369. doi:10.1016/j.jasrep.2016.09.013

18. Tryon CA, Peppe DJ, Faith JT, Van A, Nightingale S, Ogondo J, et al. Archaeological Research in Africa Late Pleistocene artefacts and fauna from Rusinga and Mfangano islands , Lake Victoria, Kenya. Azania. 2012; 14–38. doi:10.1080/0067270X.2011.647946

19. Dickson DB, Gang G-Y. Evidence of the Emergence of “Modern” Behavior in the Middle and Later Stone Age Lithic Assemblages at Shurmai Rockshelter (GnJm1) and Kakwa Lelash Rockshelter (GnJm2) in the Mukogodo Hills of North-Central Kenya. African Archaeol Rev. 2002;19: 1–26. doi:10.1023/A:1014490818099

20. Bruggemann JH, Bu RT, Guillaume MMM, Walter RC, Cosel R Von, Ghebretensae BN, et al. Stratigraphy , palaeoenvironments and model for the deposition of the Abdur Reef Limestone : context for an important archaeological site from the last interglacial on the Red Sea coast of Eritrea. Palaeogeogr Palaeoclimatol Palaeoecol. 2004;203: 179–206. doi:10.1016/S0031-0182(03)00659-X

21. Yellen J, Brooks A, Helgren D, Tappen M, Ambrose S, Bonnefille R, et al. The Archaeology of Aduma Middle Stone Age Sites in the Awash Valley , Ethiopia. PaleoAnthropology. 2005;10: 25–100.

22. Tryon CA, Roach NT, Logan MA V. The Middle Stone Age of the northern Kenyan Rift : age and context of new archaeological sites from the Kapedo Tuffs. J Hum Evol. 2008;55: 652–664. doi:10.1016/j.jhevol.2008.03.008

23. Shea JJ. The Middle Stone Age archaeology of the Lower Omo Valley Kibish Formation: Excavations, lithic assemblages, and inferred patterns of early Homo sapiens behavior. J Hum Evol. 2008;55: 448–485. doi:10.1016/j.jhevol.2008.05.014

24. Maíllo-Fernández JM, Solano-Megías I, Mabulla AZP, Arriaza MC, Bower JFR. Lithic Technology at Loiyangalani, a Late Middle Stone Age Site in the Serengeti, Tanzania. African Archaeol Rev. 2019;36: 291–315. doi:10.1007/s10437-019-09340-2

25. Blinkhorn J, Grove M. The structure of the Middle Stone Age of eastern Africa. Quat Sci Rev. 2018;195: 1–20. doi:10.1016/j.quascirev.2018.07.011

26. Tryon CA, Faith JT. Variability in the Middle Stone Age of Eastern Africa. Curr Anthropol. 2013;54 (S8): S234–S254. doi:10.1086/673752

27. Tryon CA. The East African Middle/Later Stone Age transition and cultural dynamics of the Late Pleistocene. Evol Anthropol. 2019; 267–282. doi:10.1002/evan.21802
